# Feedback practices in undergraduate clinical teaching in Sri Lanka - a qualitative study

**DOI:** 10.1186/s12909-024-05556-2

**Published:** 2024-05-22

**Authors:** Sivapalan Sanchayan, Asela Olupeliyawa, Madawa Chandratilake

**Affiliations:** 1 Medical Education Unit, Faculty of Medicine, University of Jaffna, Jaffna, Sri Lanka; 2 Department of Medical Education, Faculty of Medicine, University of Colombo, Colombo, Sri Lanka; 3 Department of Medical Education, Faculty of Medicine, University of Kelaniya, Ragama, Sri Lanka

**Keywords:** Clinical education, Medical education, Undergraduate clinical training, Video ethnography, Culture

## Abstract

**Background:**

Feedback is integral to medical education, enabling students to improve their knowledge, skills, and attitudes. Feedback practices may vary according to prevalent cultural and contextual factors. This study aimed to explore how feedback is conceptualized and practised in the clinical education of medical students in Sri Lanka.

**Methods:**

The study was conducted in three medical schools and affiliated hospitals that represent the cultural diversity of Sri Lanka. Purposive sampling was utilized to recruit clinical teachers and students who would provide rich information for the study. The study had three components: an observation study, interviews with clinical teachers and focus group discussions with clinical students. During the observation study, video recording was used as a data collection tool to observe feedback in real-life clinical teaching/learning settings. A constructivist grounded theory approach was adapted for analysis to explore current practices and perceptions inductively.

**Results:**

Feedback was conceptualised as spontaneous unidirectional provision of information for the improvement of students. It was often provided in public settings and in student groups. Error correction was the primary focus of feedback, but both teachers and students desired a balanced approach with reinforcement and reflection. Although the direct approach to corrective feedback was found beneficial for student learning, participants agreed that harsh feedback was to be avoided. The hierarchical culture and lack of programmed feedback in the curricula influenced feedback practices, suggesting the need for modification.

**Conclusions:**

This study highlighted feedback practices in the local context, emphasizing the need to address the hierarchical gap in clinical settings, balance reinforcement and correction, and promote dialogue and reflection in the feedback processes. The findings will help clinical teachers from both the global south as well as the global north to recognize cultural and contextual differences in providing feedback.

## Background

Feedback is integral to medical education, enabling students to improve their knowledge, skills, and attitudes to practice as future doctors [1, 2]. Expert feedback is crucial in identifying strengths, areas for improvement, and facilitating growth. Feedback practices are influenced by contextual factors and learning culture [3–5]. Cultural influences affect feedback seeking and provision [6, 7].

Hofstede [8] provides a useful framework to situate the impact of culture on feedback. Sri Lankan society, like other South Asian countries, is hierarchical and collectivistic [9], valuing societal and workplace hierarchies. In contrast, Western countries such as the UK, USA, Australia, and Canada, where literature on feedback is mostly contextualised in, exhibit low power distance and individualism [9]. These cultural variations impact feedback engagement. It is postulated that Western societies are low-context cultures, focusing on specific criteria-relevant information, while high-context cultures such as Sri Lanka interpret feedback cues from nonverbal behaviours, settings, and actor status [10]. Hierarchical cultures may accept status differences but resist supervisors’ influence and be less trusting of their feedback [10, 11]. Understanding the existing feedback culture is essential for effective interventions.

Despite advancements, feedback effectiveness remains a concern [12]. To address this, student feedback literacy [13] and teacher feedback literacy [14] concepts have been proposed. Student feedback literacy involves making sense of feedback information to enhance learning strategies [13]. Teacher feedback literacy includes designing feedback processes to facilitate student uptake and foster feedback literacy, encompassing design, relational, and pragmatic dimensions [14]. Exploring feedback practices in the local context will support teachers in addressing the pragmatic dimension of teacher feedback literacy and may subsequently contribute to student feedback literacy.

In this context, this study aimed to evaluate feedback practices in clinical teaching of medical students in Sri Lanka.

## Methodology

### Context and setting of the study

All Sri Lankan medical schools deliver 5-year undergraduate medical education programs. Students are admitted to these medical schools based on national high school examination scores. Faculties of Medicine Universities of Kelaniya, Jaffna and Colombo were selected to represent diverse ethnicities of the country (and the likely language used in student–student and patient-doctor communication) and range of academic merit. Faculty of Medicine, Colombo is in the capital (Colombo, Western Province) of Sri Lanka and receives students from all over the country who score within the top 10% of students selected for Medicine at the national high school examination as well as students from the western province. Faculty of Medicine, Kelaniya is also in the Western Province. It was established more recently than Colombo Medical School and receives students mostly from the Western Province. The ethnicities of Sri Lanka consist of Sinhalese (74.9%), Tamils (15.2%) and Muslims (9.3%) and other minorities (0.4%) [15] and a higher proportion of students from these two faculties are Sinhalese (about 75 to 90%). Faculty of Medicine, Jaffna is in the Northern Province. A higher proportion of the students there are Tamil (about 60%).

### Study design

Feedback is a process of communication and therefore is a socially constructed phenomenon [16]. This study utilized a social constructionist epistemology, which considers knowledge to be constructed through social interactions [17]. Therefore, in addition to observing feedback interactions that occur in clinical settings, the teachers’ and students’ perspectives regarding feedback were explored. Grounded theory research is exploratory and it seeks to understand the core social processes underlying phenomena of interest [18]. Constructivist grounded theory assumes that both the research process and the studied world are socially constructed through actions, and that historical and social conditions constrain these actions [19]. Therefore, we employed constructivist grounded theory [20] as the philosophical paradigm for this study. As constructivist grounded theory allows the selection of suitable data collection methods to address the research question [20], three methods were chosen for the study design: 1) observation of actual clinical teaching sessions using video ethnography; 2) interviews with clinical teachers and 3) focus group discussions with clinical students.

The observation component focused on analysing feedback practices in actual clinical teaching settings and for this we employed video ethnography [21] as a cost-effective and reliable method to capture naturally occurring activities.

Interviews with clinical teachers and focus group discussions with clinical students were aimed to gain further insights into participants’ lived-in feedback practices. These methods were aimed at facilitating participants to construct meaning in relation to their feedback practices as outlined below. We chose to conduct in-depth interviews with clinical teachers as they are well experienced, and more likely to elucidate their practices than the students. We considered that the teachers might be more comfortable sharing their experiences freely in individual interviews rather than in a group of their peers [22, 23]. 

We chose to conduct focus group discussions with students as they would help to identify representative experiences of other students, capture feedback practices of a wider range of their teachers [22, 24] and generate a wider range of responses than individual interviews [24]. We considered that students may be reluctant to talk about feedback interactions freely as they involve their clinical teachers and that discussing feedback in a group of peers would make them more communicative as their experiences could be similar to one another [22, 24, 25], while comments from one participant may trigger similar and divergent responses from the others [22] within a relatively short time. In each focus group at the different medical schools the participants were from the same year ensuring homogeneity and facilitating them to express their views without the undue influences of seniority amongst them [22].

### Sampling strategy

For all the studies purposive sampling was employed to recruit teachers and students who could provide rich information regarding feedback. For the observation study, a total of seven clinical teachers, one from the Medicine and Surgery departments in each faculty, and one from the Family Medicine department were selected (six male and one female). These teachers were selected based on having more than 10 years of experience in clinical teaching as faculty.

Each teacher and students’ group were observed for a maximum of 2 weeks and various activities involving feedback sessions were video recorded i.e. bedside teaching in the ward, clinic sessions, and classroom teaching. One to five feedback instances of each activity were recorded with each teacher. Feedback sessions were defined as extended communication sequences between teachers and students that included feedback about the knowledge, skills or behaviours of the students [26].

We planned to interview clinical teachers from all three medical schools with at least one teacher each from each of the disciplines involved in clinical teaching. Similar to the observation study, these teachers were selected based on having more than ten years of experience in clinical teaching as faculty.

We planned to have focus group discussions in each medical school with students from years three to five (the clinical phase of the programs), and recent graduates. Students were selected using snowball sampling (the student representatives of each cohort assisted in their recruitment) and were purposefully chosen to represent the gender balance and varying academic performances.

### Data collection instruments and procedures

While recording the clinical teaching activities for the observational component of the study, we aimed to mitigate the Hawthorne effect, whereby participants may alter their behaviour when they are aware of being observed [27]. To minimize disruption and ensure discrete observation, we utilized a compact GoPro Hero 5 camera. The identified feedback sessions were transcribed using established conventions developed by Gail Jefferson, as presented by Heath et al. [21] (Table 1).

**Table 1:** Conventions used to transcribe the video and audio data

**Transcription symbol**	**Definition/explanation**
(0.5) Or (2)	Numbers in brackets indicate intervals in a stream of talk, in seconds
[	A square bracket connecting the talk of different speakers shows overlapping talk beginning. The overlap is with the talk above it
]	A square bracket connecting the talk of different speakers shows overlapping talk ending
?	A question mark is used for rising intonation
-	A single dash is used when the utterance is cut off
(word) or ()	Words placed within parentheses offer a possible but uncertain hearing of the talk
(())	Double parentheses offer extra descriptions, often of actions (e.g. laughter, coughing, pointing etc.)
there	Underlining shows where an utterance, or part of an utterance, is emphasized

Interviews and focus group discussions were guided by semi structured guides developed based on the objectives of the study. Audio of both were recorded and transcribed verbatim. All three components of the study were completed in one medical school starting with the observations followed by the interviews and focus group discussions before proceeding to the next medical school.

### Analysis

Throughout the analysis phase, we adhered to the steps outlined by Charmaz [20] for constructivist grounded theory analysis. Observations, interviews and focus groups were completed at each school before proceeding to the next. Initial coding was commenced as soon as the first observation, interview and focus group discussion were conducted enabling analysis and data collection to proceed parallelly and inform subsequent data collection. The most significant or frequent earlier codes were used for focused coding to sort, synthesize, integrate, and organize the data [20]. While interview and focus group data were initially coded separately to facilitate the capture of diverse perspectives, similar and connected codes and categories were identified and integrated as analysis progressed. The categories identified in the interviews and focus groups provided the focal points for the analysis of the observational study. In addition, integration was also achieved by looking for explanations for observed behaviours in the interview and focus group data and vice versa.

According to Charmaz [20], theoretical codes specify possible relationships between categories that were developed during focused coding. In this study, theoretical coding was used to identify how the participants conceptualised feedback and the factors affecting feedback. Theoretical sampling is looking for data that clarifies emerging theories further [20]. The initial sampling was adequate to explore feedback in the local context and the need for further theoretical sampling was not identified. As we progressed through the data collection and analysis, data and codes within all three components of this study were compared with each other to refine the codes and categories (constant comparative analysis). To ensure the reliability and validity of our coding process, the coinvestigators engaged in discussions to reach consensus on the codes and categories.

### Reflexivity

The researchers engaged in reflexivity to be aware of preconceptions and how they may affect the design and data collection of the study [28]. The principal investigator (S.S.) is a medical graduate from Sri Lanka who had undergone undergraduate training in similar settings, and had some experience in clinical teaching in Surgery. The other two researchers (A.O. and M.C.), medical graduates from Sri Lanka with 15–20 years of expertise as medical educators, had completed their doctoral studies on clinical training and professionalism in Australia and the United Kingdom. Our collective backgrounds helped us to understand feedback practices in the local context and globally, and look at the data more objectively. All the decisions that were taken during the research design, implementation and analysis were discussed between the researchers. In addition, triangulation between all three data sources was done during the integrative analysis of the three components of the study.

### Ethical considerations

Informed written consent to participate and to record audio (for all three components) and video (for the observations) to use in this study was obtained from all the participants before each component of the study. Ethics approval for this study was obtained from the Ethics Review Committee at the Faculty of Medicine, University of Kelaniya, Sri Lanka (P/114/05/2018). We also obtained permission to conduct the study from the three participating medical schools and relevant hospitals.

To address perceived risks to confidentiality in video recording of data, the principal investigator who conducted all the data collection met with the potential participants (both teachers and students) well in advance to explain the purpose of the study and data collection method. It was made clear that only teaching learning activities involving feedback will be recorded and any sensitive situations, aspects of patient management or other ward, theatre or clinic activities would not be recorded. Assurances were made that when publishing the data arising from the analysis, all identifying features would be anonymised. When transcribing the videos, code names were provided to all the participants and the places of recording. Even though participants were offered an opportunity to view the recordings and remove any if they deemed necessary [29], no one made such a request.

## Results

We recorded a total of 31 feedback sessions using video as a means of documentation, amounting to 413 min of observation (Table 2). Of these sessions, 11 were of bedside teaching in the ward, eight were of clinic sessions, and two were of classroom teaching.

**Table 2:** The list of recorded video clips

**Teaching Instance No**	**Duration**	**Teacher**	**Context**	**Number of students**
1	19.00	T1	Surgery Ward	24
2	35:03	T1	Surgery Clinic	5
3	17:06	T1	Surgery Clinic	5
4	4:07	T1	Surgery Clinic	5
5	7:06	T1	Surgery Clinic	5
6	17:21	T1	Surgery Clinic	5
7	65:00	T1	Surgery Ward	24
8	7:24	T2	Medicine Ward	18
9	9:38	T2	Medicine Ward	18
10	7:58	T2	Medicine Ward	18
11	11:51	T2	Medicine Ward	18
12	06:10	T3	Surgery Clinic	1
13	02:26	T3	Surgery Clinic	1
14	9:18	T3	Surgery Clinic	1
15	8:10	T4	Medicine ward	22
16	7:16	T4	Medicine Ward	22
17	9:21	T4	Medicine Ward	22
18	24:31	T4	Teaching room	45
19	44:36	T5	Teaching room	31
20	5:38	T6	Surgical Clinic	14
21	5:21	T6	Surgical Clinic	14
22	5:03	T6	Surgical Clinic	14
23	6:08	T6	Surgical Clinic	14
24	11:22	T6	Surgical Clinic	14
25	8:36	T6	Surgical Clinic	14
26	7:03	T6	Surgical Clinic	14
27	17:10	T6	Surgical ward	14
28	22:07	T6	Surgical ward	14
29	9:26	T7	Family Medicine Clinic	1
30	9:03	T7	Family Medicine Clinic	2
31	11:33	T7	Family Medicine Clinic	1

For the interviews, saturation was achieved with thirteen clinical teachers from the Surgery (*n* = 3), Medicine (*n* = 3), Paediatrics (*n* = 3), Obstetrics and Gynaecology (*n* = 1), Psychiatry (*n* = 2) and Family Medicine (*n* = 1) departments (ten male and three female teachers) which lasted for a combined duration of 284 min.

For the focus groups, saturation was achieved with eight discussions (six with students from years three to five, and two with recently graduated students). Sixty-five students (27 male and 38 female students) took part. Each group had five to eleven students. The focus group discussions with students had a combined duration of 466 min.

**Fig. 1:**
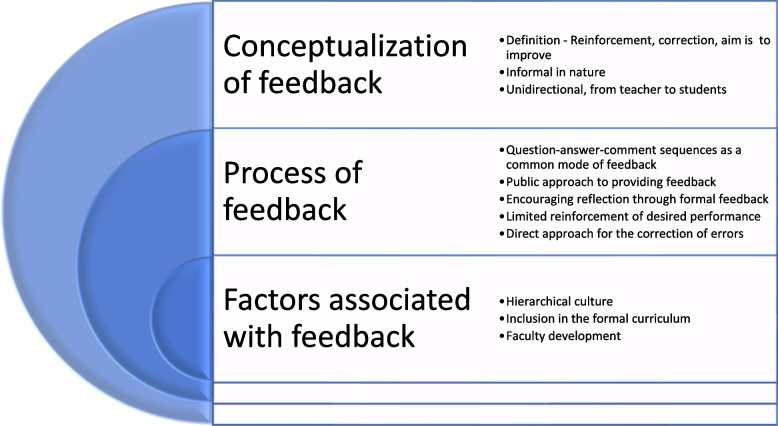
A comprehensive view of the findings of this study

Figure 1 shows a comprehensive view of the findings of this study. We present the data in three categories: conceptualization of feedback, the process of feedback and the factors affecting feedback. In the results presented below, “T” denotes teacher and “S” denotes student. Extracts from observation transcripts are in Tables, while quotes from interviews and focus groups are in boxes.

### Conceptualization of feedback

Both teachers and students conceptualised feedback as reinforcing desirable performances and correcting undesirable performances with the aim of improving the students’ performances in the future (Table 3 - FQ4). Both groups felt that the main function of feedback was pointing out the errors so that they can be corrected (Table 3 – IQ8). Feedback in the local context was not considered as a formal requirement, often occurring in a spontaneous informal manner. Although both teachers and students expressed their preference for feedback to be a discussion, in practice, it was often unidirectional.

**Table 3:** Quotes regarding conceptualization of feedback

*FQ4: (Feedback includes) “The negative feedback that we get when we do something wrong and positive feedback that we receive when we do something well. Both are helpful in opposite ways.” (S33, Graduate, Faculty C, Male)*
*IQ8: “Normally only when they do a wrong thing, we jump on them or at least tell in a good way. If they do a positive thing, we tend to ignore that. Not consciously. You have done the correct thing. Why should I say that you are correct?” (T7, Male family physician)*

### The process of feedback

During the observations, interviews and focus group discussions, the following areas were identified as key elements in the process of feedback: question–answer-comment sequences as a common mode of feedback; “public approach” to providing feedback in groups; encouraging reflection through formal feedback; limited acknowledgement and/or reinforcement of desired performance and frequent use of a direct approach for the correction of errors (Fig. 1).

#### Question–answer-comment sequences as a common mode of feedback

Question–answer-comment sequences [30, 31] were prevalent as a feedback mechanism, featuring a repetitive triple utterance structure. This structure included a question posed by the teacher, a corresponding answer provided by the student, and an evaluation of the answer by the teacher, which served as feedback. As an illustration, in the surgical clinic, a noteworthy discussion took place regarding a patient with inflammatory bowel disease. Table 4 provides a snapshot of this discussion.

**Table 4:** Discussion regarding a patient with inflammatory bowel disease

**Turn**	**Party**	**Utterance/Action**
1	T1:	This is a 39 year old female. Her main complaints are ((counts on fingers)) right lower quadrant pain, watery stool for 3 days and weight loss. She has lost 20 kilos over 6 months. What are the possibilities? (3) ((writes on the clinic note book of the patient, then looks up)) So one cause that has been suggested is Chron’s…
…		
7	T1:	Why do Chrohn’s patients have weight loss?
8	S2:	Poor absorption
9	T1:	((Nodding)) mal absorption (2)
10	S2:	Poor (intake)
11	T1:	Why do they have poor (Intake)?
12	S2:	They get loss of appetite
13	T1:	((Shaking head)) not really
14	S2:	Due to abdominal pain, difficult to eat
15	T1:	Ok, if there is pain or if meals precipitate pain, that might inhibit food intake. Appetite is usually preserved unless it’s an acute exacerbation. What else?
16	S3:	Cytokines, the inflammatory process
17	T1:	((Nodding)) the inflammatory process. It’s a chronic inflammatory process isn’t it. The inflammatory process itself, as you said the inflammatory cytokines will cause (1) ((nods head slightly)) what?
18	S3	Diarrhoea

Question–answer-comment sequences were observed both in the presence of patients (in the ward or clinic) and in their absence (during clinical discussions in learning rooms). As identified in the interviews, teachers viewed questioning students as an opportunity for providing feedback, while students identified it as a primary form of feedback (Table 5).

**Table 5:** Quotes regarding the question-answer-comment sequences

*IQ5: “One way I ensure I give some amount of feedback is I ask every student a question. I go around in groups.” (T1, Male Surgeon)*
*FQ3:—“During the ward classes, they (the teachers) ask questions about the conditions and depending on the answers that we give, we get feedback.” (S63, Fourth year, Faculty A, Female)*

#### “Public approach” to providing feedback in groups

Clinical teaching occurred in various settings, including wards, teaching rooms, outpatient clinics, and operating theatres. However, due to resource constraints, feedback was often delivered in crowded environments alongside other students, staff (such as medical officers, nurses, and attendants), and patients. The size of the student groups varied but was relatively large (15–40) in most instances. There was minimal regard for the privacy of students receiving feedback in these settings. Many teachers emphasized the importance of maximizing teaching opportunities by adopting a “public approach” to providing feedback, which students generally viewed as advantageous (Table 6 - IQ7, FQ14). However, some students highlighted the drawbacks of this lack of privacy, including challenges in discussing feedback in detail (Table 6 - FQ13) and potential patient reluctance in future interactions (Table 6 - FQ18).

**Table 6:** Quotes regarding the public approach to feedback

*IQ7: “I think group feedback is also feedback. So even though I am addressing one person, I know a lot of the others also have similar thought process.” (T1, Male Surgeon)*
*FQ14: “Sometimes, it is not always about individualized comments. It can be a generalized comment as well to learn the common mistakes… It could be an eye opener in the way that they have thought about the problem.” (S11, Fifth year, Faculty B, Female)*
*FQ13: “When we are criticized (given feedback) privately, we have the chance to ask questions… If we are given criticism (given feedback) in public, it would be a bit difficult for us to ask them back as to what I should do.” (S16, Third year, Faculty C, Male)*
*FQ18: (When receiving critical feedback in public) “Sometimes, we can’t even follow up the patients because they stop giving information. Even sometimes, they tell the neighbouring patients as well.” (S41, Graduate, Faculty A, Male)*

#### Encouraging reflection through formal feedback

In formal feedback sessions in a teaching room environment without a patient, which were less frequent than question–answer-comment sequences, the student was encouraged to analyse and reflect on their own performance. As an example, the following interaction took place during a practice long case discussion (which is a component of the clinical assessment for medical students in Sri Lanka) on a patient with prolonged fever (Table 7).

**Table 7:** A case discussion on prolonged fever

**Turn**	**Party**	**Utterance/Action**
1	T5:	Overall, how do you rate your performance? (2)
		Well or not well (4)
		Are you eligible to pass?
2	S6:	[Yes ((nods head to show agreement))
3	Other students	[Yes
4	T5:	Are you eligible to become an HO (House Officer)?
5	S6:	Yes ((nods head to show agreement))
6	T5:	Which part did you think that you did not do well? (3) History (.) Examination (.) formulating a differential diagnosis or formulating investigations (.) which part did you think you did not do well?
7	S6:	Differential diagnosis
8	T5:	Differential diagnosis ((Nods)). You got that it was a prolonged febrile illness. You thought of TB. And thought of something else in the hepatobiliary system (2). No harm (). Now how many marks would you allocate?
9	S6:	50
10	T5:	Anybody more than 50 ((looking at other medical students and smiling))
11	S7:	55
12	T5:	Don’t do the systems review like a second-year medical student here. After the history of presenting complaint, get the differential diagnosis and then go back and ask the system review related to the differential diagnosis.

During this feedback exchange, the teacher initiated the conversation by asking the student to assess his own performance and whether he believed he had performed well or not. However, the student remained silent, indicating hesitancy or an inability to freely express his self-evaluation. To encourage reflection, the teacher further inquired about the student’s eligibility for a passing grade, the marks he would assign himself for this performance, and his readiness to assume the role of a house officer (intern). In the focus group discussions, students expressed the belief that feedback that fostered reflection was beneficial (Table 8). However, it appeared that encouraging reflection was at a basic level and not fully utilized.

**Table 8:** Quotes regarding encouraging reflection

*FQ42: “Sometimes the consultant asks us ok, now tell me what you did good. What did you do wrong? So, I have a reflection on my performance. That is a better way of getting feedback.” (S9, Fifth year, Faculty B, Male)*	

#### Limited acknowledgement and/or reinforcement of desired performance

During the observations, when students performed according to the teacher’s expectations or provided correct answers, the teachers responded through brief verbal affirmations (“yes”, “good”, or repeating the correct answer) and through nonverbal cues such as nodding. The feedback interaction depicted in Table 9 took place after a case discussion in the form of a practice session for a long case. In this session, rather than addressing the student who presented the case directly, the teacher addressed the entire class. In this example, the teacher highlighted the aspects of the performance that met his expectations. Following the session, the teacher informed the researcher that this was considered a good performance and that opportunities to acknowledge such performances were rare. He further mentioned that he typically maintained a deliberately challenging stance towards students to motivate them to continue their learning.

**Table 9:** Example of reinforcing a desired performance

Line	Utterance
1	Right. ((looking at the other students in the room)) let me just analyze this
2	discussion, ok. … She did it absolutely well. … History was perfect. Findings were
3	correct. … Examination was good…what I wanted in this situation is that he is an old
4	gentleman with ankle edema, who does not have heart failure. I wanted you to
5	specifically identify that this was not anything else but renal. She very clearly did that.
6	That’s all I wanted. If she can do that that’s basically 70. The way she presented she
7	got up to 80. …

Teachers perceived that the local context lacked adequate recognition of students’ positive performances (Table 3 - IQ8). Students also shared their perception of an imbalance between appreciation for correct performances and highlighting mistakes. Positive performances were often not acknowledged or commented upon (Table 10 - FQ20). Students typically interpreted the absence of negative comments from teachers as an indication of acceptable performance (Table 10 - FQ22). While most teachers recognized the importance of acknowledging positive performances, they were concerned that students might overlook necessary corrections if the positive aspects were acknowledged at the outset (Table 10 - IQ11).

**Table 10:** Quotes regarding acknowledgement of desired performance

*FQ20: “What I have seen happening is that if its negative comments, it is very much negative. Whereas if a student performs well, the positive comments, the encouragement that they (the teachers) give the student is rather lacking. There is a drastic gap between the positive comments and the negative comments.” (S39, Graduate, Faculty A, Male)*
*FQ22: “About half the time, when we do something satisfactorily, they don’t say anything. They (just) don’t say anything negative.” (S32, Graduate, Faculty C, Female)*
*IQ11: “When you start of saying ok, you have done very well, and an important mistake was made—if you go around and round talking about the good things, the student will go home with a false idea…. If they do something really bad, I would say tell it outright at the beginning itself.” (T11, Female paediatrician)*

#### Frequent use of a direct approach for the correction of errors

Teachers often prioritized correcting the mistakes made by students, considering it the primary purpose of feedback. Teachers employed different strategies to highlight perceived errors. These strategies included providing direct corrections (Table 11), indirect corrections, and creating opportunities for self-correction. The teachers demonstrated thoughtfulness in delivering corrective feedback to students. While they occasionally employed directness, they were mindful of avoiding harshness.

**Table 11:** An example of direct correction

**Turn**	**Party**	**Utterance/Action**
1	S8:	No history of significant medical problems
2	T1:	Don’t say significant. What is significant or not? ((Stops writing and looks at the student))
3	S8:	Evidence for having any medical conditions
4	T1:	If you say significant, what is significant to you and what is significant to me is different. So you have to say not diabetic, not hypertensive and so on.

Teachers often avoid giving direct negative comments by employing hedging strategies. Instead of explicitly stating, “no, the patients do not…”, they would shake their head and say “not really”. They utilized extended question–answer sequences and reoriented questions to provide students with opportunities to correct themselves.

During interviews and focus group discussions, it became evident that feedback is often delivered directly without considering the students’ feelings or potential embarrassment (Table 12** - **IQ 22). Harsh corrections are also common, as teachers believe they are necessary for student learning (Table 12** - **IQ15). While the direct nature of correction presents a challenge for students, it offers advantages. It provides a clear understanding of performances that fall short of expectations and need improvement (Table 12** - **FQ29). Many teachers believed that feedback should be delivered in a less direct and more supportive manner to foster students’ active participation and confidence in teaching and learning activities (Table 12** - **IQ19). Students also express that corrective feedback is most beneficial when delivered in an acceptable manner, using neutral language and tone without resorting to harsh words or actions (Table 12** - **FQ32). They find inflammatory language unnecessary and unproductive.

**Table 12:** Quotes regarding correction of errors

*IQ22: “If I ask the student to examine a patient, I’ll be more concerned with teaching the eliciting of a clinical sign. Whether the clinical sign is elicited properly or not… You didn’t do it correctly. This is the correct way.” (T7, Male family physician)*
*IQ15: “For some students, this (negative feedback) is a thing they have learnt, and lot of very senior and experienced teachers seem to tell me, in these (local) settings, you have to be authoritarian and there has to be some punishment. You should have some stick behind saying you are going to be repeated if you don’t learn this and come next time. And some students seem to want that kind of relationship. Or feedback in those authoritarian, strong, clear terms.” (T12, Male psychiatrist)*
*FQ29: “Negative feedback helps us to overcome any mistakes we have made or any shortcomings that we have on our side and to take steps to be a better person.” (S7, Fourth year, Faculty B, Male)*
*IQ19: “I think if you start off by saying this is not how you do it, you put them on a negative footing and then they get scared. If they get scared, they don’t say a thing. Sometimes to get them to talk, you have to show that you are not going to pounce on them and eat them up if your asking questions.” (T8, Female paediatrician)*
*FQ32: “It’s not the comment I think it’s how the comment is expressed. That is the most important thing … Negative feedback should be given. But in a good manner. Not in a harsh manner.” (S6, Fourth year, Faculty A, Male)*
*FQ84: “Sometimes I’ve seen in my peers how they have all become silenced because of such adverse feedback or rather adverse ways of giving feedback. and because of that, I feel that people don’t actually talk.” (S61, Fourth year, Faculty A, Female)*
*FQ85: “So now I avoid taking important histories because I am afraid of getting scolded. I will ask, please I don’t want any important patients because I will have to present it.” (S59, Fourth year, Faculty A, Female)*
*FQ78: “After passing the Advanced Level (examination in school), we came here with confidence. And after entering the faculty, mainly after the clinical stream, that confidence is let down bit by bit, until we feel we are not fit. We are not even confident enough to answer questions. We doubt ourselves that much.” (S60, Fourth year, Faculty A, Male)*
*FQ82: “I think there would be a significant percentage of students who will admit that … their openness, their positive attitude and their spontaneity is somewhat or greatly reduced by this kind of environment. So they are not forward anymore.” (S42, Graduate, Faculty A, Male)*

The findings suggest that these practices have detrimental effects on students’ confidence and their willingness to seek feedback and engage in learning (Table 12 - FQ84). Constant negative feedback, without recognition of positive aspects in their performance, and harsh corrections delivered in the presence of patients and other staff, contribute to this outcome. As a result, some students become reluctant to answer questions, volunteer for tasks, and present patients (Table 12 - FQ85). Such behaviours are not conducive to a supportive learning environment.

When students perceive that teachers are disinterested in their personal growth, maintain excessive power distance, or adopt a harsh approach, they tend to disregard the feedback provided. Constant exposure to negative feedback has a detrimental impact on their confidence (Table 12 - FQ78) and diminishes their enthusiasm for learning medicine (Table 12 - FQ82).

In summary, students appreciate a direct approach to error correction when it aims to improve their performance. Despite the negatively tilted feedback prevalent in the learning environment, students tend to tolerate it, although they express a desire for the recognition of their positive performance aspects.

### Factors influencing feedback in Sri Lanka

The key factors identified by the study that influenced feedback were the hierarchical nature of the teacher student interaction, lack of inclusion of feedback in the formal curriculum and the need for faculty development.

#### Impact of the hierarchical nature of teacher-student interaction on effective feedback discussions

Teachers in local settings acknowledge the hierarchical distance between them and students (Table 13 - IQ30, 31). This gap leads to unidirectional and prescriptive feedback, limiting student participation in discussions. While teachers recognize the benefits of narrowing the gap, they believe that some distance and authority are necessary to ensure student compliance and task completion (Table 13 - IQ31).

**Table 13:** Quotes regarding the hierarchical nature of the interactions

*IQ30: “You think you are a consultant; you are a big shot. ((laughs)). When you want subordinates, it’s not a team. That is one negative aspect in feedback. The hierarchical structure. It also inhibits the students from asking feedback. They are scared.” (T11, Female paediatrician)*
*IQ31: “In our hierarchy, students think that they have a gap between us and them. They are really scared. When they make a mistake, we also subconsciously point fingers and we are harsh and they get scared. So, we need to reduce that gap. Then only they can freely come and (talk to) us. We have to be accessible to them. One fine day all of us are colleagues. So, the gap should not be there. But at the same time if you are very close also, sometimes, they don’t do what we say. You have to have a sort of small gap and they should know that what we say is for their benefit. They must learn, they must do what we tell them. At the same time the gap should not be very large.” (T2, Female physician)*
*FQ68: “From grade 1, we are trained to treat the school teachers and adults as superiors, seniors. The same attitude is seen in the medical school.” (S39, Graduate, Faculty A, Male)*
*FQ69: “When we need to clarify things, because of the fear and the gap that exists between us and him we will not ask. Then his expectations will not be transparent to us. And our problems will not be transparent to him. So that there will be a big mess all the time.” (S16, Third year, Faculty C, Male)*
*FQ70: “The opportunity to (discuss wrong answers) is also less if the distance (between teacher and student) is more. Say the student gave a wrong answer and he may want to discuss about it. Why he gave that answer. So that opportunity is not there. People simply accept it.” (S49, Graduate, Faculty A, Male)*
*FQ71: “There are students who when you get feedback from a consultant who is distant to you, then you just ignore it. But when a close father figure or mother figure like consultant give something, they tend to correct it. Because you don’t want to upset that person. You know that he is telling that for your own good. But if you are more distant, you ignore it. Because you know that doesn’t matter.” (S45, Graduate, Faculty A, Female)*
*FQ72: “It is also the hierarchical nature we have in our profession. We rarely see even a postgraduate registrar or senior registrar having an individual kind of questioning with the consultant. So, I don’t think they can expect us as undergraduates to do the initiating (of feedback) when the situation is like that.” (S7, Fourth year, Faculty B, Male)*

Students attribute this hierarchical gap not only to the medical education system’s hierarchy (Table 13 - FQ68) but also to their upbringing, where they were taught to maintain a respectful distance from teachers.

The presence of this hierarchical gap hinders effective communication and learning, as teachers and students do not engage in free and equal dialogue (Table 13 - IQ30, IQ31, FQ69). This hampers open communication and useful feedback, preventing students from seeking clarification (Table 13 – FQ69), asking questions, reasoning (Table 13 - FQ70), volunteering, and answering questions due to the fear of making mistakes.

When students perceive teachers as distant, they may underutilize the received feedback (Table 13 - FQ71). Moreover, the hierarchical nature discourages students from volunteering or seeking feedback in front of teachers, and they rarely observe their seniors, including residents, approaching teachers for feedback (Table 13 - FQ72).

In summary, the hierarchical nature of the teacher-student interaction was perceived as disadvantageous for effective feedback discussions.

#### Lack of inclusion of feedback in the formal curriculum

Feedback is built into clinical teaching by convention and not by explicit curricular demands. The curricula do not mention that the students should seek feedback or that the teachers should provide feedback (Table 14 – IQ4). Feedback is naturally provided by teachers when they request students to perform certain clinical tasks and then provide corrections (Table 14 – IQ4).

**Table 14:** Quotes regarding the lack of inclusion of feedback in the formal curriculum

*IQ4: “There are no scheduled time for us to give feedback to the students. This should be incorporated into the timetable. There should be time slots allocated. Because there is no time, it’s all kind of offhand. It’s not programmed feedback. I’m not sure how far they will take it for the learning process. If it is programmed and we say, you are having the short case now, and you will get feedback on that, then that will go as programmed feedback into the curriculum.” (T11, Female paediatrician)*
*IQ32: I have found that the Mini CEX is really good to give the students feedback—on their knowledge, how they behave, how to approach a patient, see the physical signs, to interpretation. And I have found that students find it as one of the most useful aspects of our learning program. (T1, Male Surgeon)*
*FQ73: (on receiving feedback during a Mini CEX) For me that was really constructive. The environment, the words he (the consultant) used, the way he presented it was not threatening at all. He waited until I had finished the examination and he mentioned my good points first. I felt like I did something good. And then he came up with the negative comments in a very friendly and non-threatening manner. (S47, Graduate, Faculty A, Female)*

Feedback is facilitated in clinical teaching in Sri Lanka by the presence of long cases and short cases in the examinations. This necessitates the teachers to prompt the students to practice these formats during the rotations and then correct them so that the students could perform well at the examinations. Some teachers are also mindful of the future practice needs of the students and include this in the feedback.

Both the teachers and students feel that making feedback a conscious effort may improve the quality of feedback and the uptake by students (Table 14 – IQ4). In surgery, in faculty A, final year, the teacher and students had practiced Mini clinical examinations (mini CEX, a form of workplace-based assessment) in which feedback was provided based on Pendleton’s rules [32]. Both the teachers and students found those feedback experiences refreshing and effective (Table 14 – IQ32, FQ73).

#### Need for faculty development

During the interviews, the teachers indicated that training clinical teachers to provide feedback is essential to improve feedback practices (Table 15 – IQ34). In the Sri Lankan context, many clinical teachers were not advised on how to provide feedback and they had not conceptualized that many interactions that they have with students could be seen as feedback. In addition, the students had indicated that sometimes teachers had a poor attitude towards feedback (Table 15 - FQ67). The participants indicated that faculty development may be helpful in these regards.

**Table 15:** Quotes regarding the need for faculty development

*IQ34: “Raising the awareness of the importance of feedback will make people move up because we are more didactic in our approach. We just give a lecture and we just give instructions. But don’t spend much time giving feedback.” (T10, Male psychiatrist)*
*FQ67: “There was a consultant who marked the feedback form (on student performance during a clinical rotation) in front of us. He seemed to make fun of the feedback form … It seems that even if there is a feedback form, some don’t get the idea of the value of feedback.” (S58, Fourth year, Faculty A, Female)*

## Discussion

The conception of feedback of the participants of this study, that feedback is information that is provided to students with the intent of their improvement, matches an operational definition of feedback in clinical settings based on a literature review [33]. Feedback as practiced in the local context was not considered a formal requirement and it often occurs in an organic informal manner. Even so, the students in this study found it useful and preferred it to receiving no feedback at all. The literature in other settings also confirm that students find both formal and informal feedback as effective [34], although more systematic approaches may improve the outcomes. Feedback was seen by the participants as information provided by the teacher rather than a discussion between the teacher and students. Another study in the Southeast Asian region which has similar cultural context also found that the teachers expressed that it was their responsibility to provide feedback to students under their supervision [6]. Our study identified the possible reasons for this in the local context as feedback focused on correction of errors, the prevalent hierarchical culture, lack of visibility of other learners such as residents seeking feedback and a lack of training in feedback.

Question–answer-comment sequences were recognized to be a common form of feedback by the teachers and students. Question–answer-comment sequences were used by McHoul [30] to analyse classroom interactions and by Rizan et al. [31] to analyse feedback in action in bedside teaching settings. Awareness of the power of the feedback that students obtain from these interactions could help to improve teacher-student interactions and student communication.

This study found that feedback in the local context is commonly provided in the presence of groups consisting of students, patients, and other healthcare staff. The size of student groups can vary from two to 20 in clinics and 20 to 40 in other settings. This group size is relatively high compared to a study conducted in the United Kingdom by Urquhart et al. [26], where students had more privacy in the workplace, either being the sole student or with one other student. In simulated settings, there were typically three to ten other students present. Limited teacher resources, such as a high staff-student ratio, contribute to these contextual differences in resource-constrained settings, despite an abundance of patient resources.

Nevertheless, this study demonstrates that providing feedback to individuals where recipient privacy cannot be ensured can still be beneficial if done appropriately, benefiting both the individual and the observing group of students. In the literature on feedback in Western settings, there is an emphasis on privacy for feedback to be effective [34], and there is little mention of the benefits of feedback to individuals in groups. Although some students in this study indicated that they avoid situations that may lead to feedback issues, many students mentioned that they have learned to engage and disregard the negative consequences of feedback during their clinical rotations. Building on other studies [35], this study also highlights the importance of providing very critical feedback away from busy clinical settings to maintain privacy and preserve student self-esteem.

During feedback sessions, the teachers in this study rarely guided students to reflect deeply on their performance. When opportunities for reflection were provided, the students viewed them positively. The prevailing culture seems to cause the students in this study to assess themselves negatively. After reflecting on their areas of poor performance, when the teachers pointed out the areas in which they did well, it boosted their confidence and self-efficacy beliefs. While most feedback guidelines encourage reflection by the feedback recipient [32, 36], there is limited empirical evidence. In an Australian study, Rees et al. [37] found that asking students to reflect on their performance was seen as sharing power in feedback relationships. This aspect of feedback appears to be highly valued in the Sri Lankan context and warrants further exploration.

In this study, both the teachers and students considered reinforcing positive performances as an essential component of feedback, as it encourages students and helps them maintain good practices. Participants in this study perceived a lack of balance between reinforcement and correction in the local context, a phenomenon noted by educators in the region [38] and identified in other studies [6, 35]. The present study identified possible reasons behind this limited reinforcement. One key reason, apparent from interviews and focus group discussions, is that reinforcing positive performance is not considered essential and useful by society in general, including the teacher and student population. This perception of reinforcement as being of little value is also found in other Asian settings [6]. Receiving reinforcement appeared to improve the confidence of the recipients in this study, as observed in other local studies [35] and in Western settings [34, 39]. Participants in this study, as well as the study by Areemit et al. [6], expressed a desire for improved provision of reinforcing information. It should be noted that when reinforcing feedback does not align with the recipient’s perception (who believes they performed poorly), they tend to disregard it [34]. This is a pitfall that needs to be avoided.

In this study the participants perceived that error correction was the primary function of feedback. Similar to other studies in East Asian cultures [6, 40], feedback was initiated when teachers identified deficits and provided corrections to the students. Feedback was often provided in a direct and sometimes harsh manner, with perceived advantages in terms of students gaining a clear understanding of their errors and the necessary corrections. Most students have learned to overlook the harsh elements and focus on the learning points. Similar to this study, students in East Asian cultures believed that although harsh feedback made them uncomfortable, it could be useful when they perceived the teacher’s intent to be improving their performance and acting in their best interest [6]. Suhoyu et al. [41] also found that students in Asian cultures believed corrections were more valuable than reinforcements. This aligns with the perceptions of students in this study. In some Western settings, a culture of politeness implicitly discouraged corrective feedback [42, 43], whereas in Sri Lanka, this was not a consideration.

While direct feedback focused on error correction was perceived positively in this study, both teachers and students expressed a desire for less harsh and more supportive feedback. Similar findings were observed in other Asian studies [6, 35]. In some Western settings, institutional reputation and teachers’ considerations of their trainees’ pedigrees hindered the provision of constructive and corrective feedback [42]. In the Sri Lankan setting, constructive feedback was hindered not by a lack of useful information but by the harsh or negative nature of the feedback.

The hierarchical nature of Sri Lankan society and the clinical learning settings creates challenges for open communication between teachers and students, resulting in feedback being perceived as unidirectional. Teachers are less likely to encourage student reflection, and students may avoid meaningful discussions and reflection even when invited. Similar findings were observed by Areemit et al. [6], where hierarchical cultures perpetuated top-down unidirectional feedback, and feedback was mostly prescriptive, with problems identified and solutions provided. Few teachers engaged students in developing action plans for future improvement. Strategies aimed at improving feedback processes in hierarchical cultures may need to focus on fostering dialogue, reflection, and action planning.

Our study highlighted that feedback was rarely mandated by curricular documents and very little guidance was provided on how feedback was to be given. Similar observations were made by other studies within the country and region [6, 35]. Both teachers and students felt that the feedback expectations must be clearer and perhaps mandated. The need for faculty development in feedback was greatly felt by the teachers and students in this study. Unsurprisingly, participants of other studies have mentioned the same [6, 35]. Many studies have suggested students need training in receiving feedback [6, 42, 44] which was mentioned by very few participants of this study. It is notable that feedback literacy is a key concept that has been gaining prominence in recent times [13].

This study has a few limitations as well as strengths. One limitation is that sampling was conducted to maximise selection of individuals who could elaborate the studied phenomena and therefore the findings may apply only to the study settings. The local conditions and methods were described so that the readers would be able to employ judgement when applying these findings to their own contexts. The teachers who were observed were experienced university teachers who were exposed to principles of teaching and learning. This sample was not representative of all the clinical teachers in the country. However, the combination of the three study methods shed light on the practices of feedback among clinical teachers in general. Lastly, applying reflexivity and providing a thick description of the explored phenomena provides adequate context for the readers to contextualise the findings.

The observation component of this study employed video recording to analyse feedback instances. This paves the way for video reflexive ethnographic studies that obtain the reflections of the participants on recorded feedback instances providing better understanding regarding feedback practices. Video reflexive ethnography could be coupled with simulation training and debriefing for interesting avenues of feedback research in more controlled environments. This study focused on clinical teachers as the source of feedback. Future studies could explore other sources of feedback that the students have access to such as other healthcare professionals, peers and electronic sources. In addition, this study highlighted how feedback was seen by the participants as information provided by the teachers. The feedback literacy of students and how they seek and utilise feedback for their improvement in the local context could be explored in future studies.

## Conclusion

This study explored feedback in Sri Lankan undergraduate clinical education, addressing a gap in the literature. The study indicates that the teachers and students have adopted certain strategies for feedback that match the existing local and institutional cultures and contexts. Some of these strategies such as utilizing feedback to an individual to inform the larger group regarding good practices and the direct approach to corrective feedback may have a beneficial effect on learning. Other less effective strategies suggest the need for medical educators to address the hierarchical gap, balance reinforcement and correction and promote dialogue and reflection. Overall, this study contributes to the literature on feedback in regional clinical education. The findings will help clinical teachers from both the global south as well as the global north to recognize cultural and contextual differences in providing feedback in an era of transhemispheric migration.

## Data Availability

The datasets generated and/or analyzed during the current study are not publicly available due to ethical considerations but are available from the corresponding author on reasonable request.
